# Influence of Antihistamines on Basophil Activation Test in Food Allergy to Milk and Egg

**DOI:** 10.3390/diagnostics11010044

**Published:** 2020-12-30

**Authors:** Eleonora Nucera, Riccardo Inchingolo, Rosario Nicotra, Manuela Ferraironi, Anna Giulia Ricci, Giuseppe Parrinello, Marilena La Sorda, Maurizio Sanguinetti, Antonio Gasbarrini, Angela Rizzi

**Affiliations:** 1 UOSD Allergologia e Immunologia Clinica, Dipartimento Scienze Mediche e Chirurgiche, Fondazione Policlinico Universitario A. Gemelli IRCCS, Medicina e Chirurgia Traslazionale, Università Cattolica del Sacro Cuore, 00168 Roma, Italy; eleonora.nucera@policlinicogemelli.it; 2 UOC Pneumologia, Dipartimento Scienze Mediche e Chirurgiche, Fondazione Policlinico Universitario A. Gemelli IRCCS, 00168 Roma, Italy; 3 UOC Microbiologia, Dipartimento Scienze di Laboratorio e Infettivologiche, Fondazione Policlinico Universitario A. Gemelli IRCCS, 00168 Roma, Italy; rosarionic@libero.it (R.N.); manuela.ferraironi@policlinicogemelli.it (M.F.); marilena.lasorda@policlinicogemelli.it (M.L.S.); 4 UOSD Allergologia e Immunologia Clinica, Dipartimento Scienze Mediche e Chirurgiche, Fondazione Policlinico Universitario A. Gemelli IRCCS, 00168 Roma, Italy; a.giuliaricci@gmail.com (A.G.R.); p.giuseppe@email.it (G.P.); angela.rizzi@policlinicogemelli.it (A.R.); 5 UOC Microbiologia, Dipartimento Scienze di Laboratorio e Infettivologiche, Fondazione Policlinico Universitario A. Gemelli IRCCS, Scienze Biotecnologiche di Base, Cliniche Intensivologiche e Perioperatorie, Università Cattolica del Sacro Cuore, 00168 Roma, Italy; maurizio.sanguinetti@policlinicogemelli.it; 6 UOC Gastroenterologia, Dipartimento Scienze Mediche e Chirurgiche, Fondazione Policlinico Universitario A. Gemelli IRCCS, Medicina e Chirurgia Traslazionale, Università Cattolica del Sacro Cuore, 00168 Roma, Italy; antonio.gasbarrini@policlinicogemelli.it

**Keywords:** basophil activation test, BAT reactivity, food allergy, antiallergic drugs, antihistamines, α-lactoalbumin, β-lactoglobulin, casein, egg white, egg yolk

## Abstract

Background: The basophil activation test (BAT) is used to improve the accuracy of food allergy diagnosis. To date, the influence of antiallergic drugs on BAT reactivity is poorly investigated. The aim of the study was to investigate if BAT results were influenced by antihistamine intake for 3 months in a cohort of patients with IgE-mediated food allergy to milk or egg. Methods: A retrospective, single-center, observational study was performed. We enrolled subjects with history of hypersensitivity reaction after specific food ingestion, positive skin prick tests and specific IgEs, concomitant allergic rhinitis, and, contraindication to the double-blind, placebo-controlled food challenge due to personal history of systemic reactions related to the ingestion of culprit food. Validated allergens (α-lactoalbumin, β-lactoglobulin, casein, egg white, and yolk) for BAT were used. Results: Thirty-nine patients with well-documented food symptoms and positive allergological workup were included in the study. BAT was positive in 29 patients. The mean percentages of CD63+ expression to specific culprit allergen did not change after the administration of drugs. Conclusions: This was the first study assessing the effects of oral antihistamines on basophil reactivity in cow’s milk and egg food allergy. Antihistamines do not interfere with BAT results.

## 1. Introduction

IgE-mediated food allergy is increasingly recognized as a growing public health burden with a prevalence ranging from 0.1% to 6.0% in Europe [1] and high morbidity and mortality rates [2]. Diagnosis of food allergy requires a combination of clinical history, skin prick tests (SPTs) and laboratory tests (specific IgE measurements, sIgE) with the suspected allergens. However, SPTs and sIgE have a high sensitivity but poor specificity, and, therefore, in case of ambiguous clinical history, it is possible to find a large proportion of false-positive results or low positive predictive values (PPVs) [3]. Therefore, the gold standard to diagnose IgE-mediated food allergy remains the double blind, placebo-controlled food challenge (DBPCFC). However, this test is potentially dangerous. In fact, in literature, it is reported that 20% to 70% of patients report allergic reactions, which are unpredictable and, in some cases, even potentially life threatening; therefore, oral food challenge must be performed under clinical supervision in specialized clinics [3].

Instead, the basophil activation test (BAT) is used to improve the accuracy of food allergy diagnosis and has been assessed in numerous studies in literature [4]. The sensitivity of this test for the diagnosis of food allergy ranges from 77% to 98%, while the specificity ranges from 75% to 100%. Therefore, BAT results could be more accurate than SPTs and sIgE [5,6,7].

The BAT consists in a flow cytometry test, which reproduces in vitro IgE-mediated hypersensitivity reactions measuring the expression of activation markers on the surface of basophils, which are upregulated through the cross-linking of IgE antibodies bound to the high-affinity IgE receptor (FcεRI).

Currently, a wide methodological heterogeneity is adopted. In fact, different basophil identification and activation markers are considered. The most common identification markers are: eotaxin CC chemochine receptor 3 (CCR3), the combination of interleukin 3 receptor alpha chain (CD123), human leukocyte antigen (HLA-DR), the combination of prostaglandin D2 receptor (CRTH-2), and the basophil-specific ectonuclease CD203c. Instead, the most common activation markers are: CD63 and the upregulation of CD203c [8,9].

Same clinical laboratories use whole blood or isolated peripheral blood mononuclear cells (PBMCs), which include basophil cells. Whole blood BAT should be performed within 4 h of blood collection, and it is possible to use ethylenediaminetetracetic acid (EDTA) or acid citrate dextrose (ACD) to prevent basophil degranulation. Moreover, different allergen stimulants can be used: crude extracts, recombinant, or purified single allergen sources [8].

The optimal efficiency of basophils’ recovery can be influenced by types of blood and stimulation buffer solutions. In fact, heparinized whole blood incubated with simulation buffer solutions with a high calcium content can potentially influence basophil stimulation [10]. On the other hand, blood samples collected in heparin tubes are considered adequate for quantification of upregulation of basophil CD203c and identification of basophil population with high levels of fluorescence intensity, with the possibility to perform tests after blood is stored for 24 h at 4 °C [11].

To date, it is known that antihistamines do not interfere with basophil reactivity, because they only block the effect of histamine, but not mediator release from basophils or mast cells [9]. Nevertheless, the influence of popular antiallergic medication on BAT reactivity is poorly investigated. The BAT is a very useful and precise method to diagnose allergy to aeroallergens (*Dermatophagoides pteronyssinus* and pollens), which is not influenced by cetirizine [12]. Moreover, no effect on test outcomes was observed at therapeutic doses of dimetindene and desloratadine in patients with hymenoptera venom allergy [9].

The aim of this study was to assess if BAT results were influenced by the use of antihistamines in a cohort of patients with IgE-mediated food allergy to milk or egg.

## 2. Materials and Methods

In a retrospective single-center study, we screened patients with IgE-mediated cow’s milk or hen’s egg allergy referred to the Allergy Unit of the Fondazione Policlinico Universitario Gemelli IRCCS in Rome. The inclusion criteria were: (1) history of hypersensitivity reaction after specific food ingestion, (2) positive SPTs, (3) specific IgEs and (4) contraindication to DBPCFC, and (5) concomitant allergic rhinitis. Exclusion criteria were concomitant therapy with oral corticosteroids, β-blocking drugs, autoimmunity disorders, severe renal, and/or cardiovascular diseases. 

Each patient was screened for food allergy, suggested by a clinical history of oral allergy syndrome (oral itching, perioral erythema, and edema of the lips, tongue, and pharynx), generalized urticaria-angioedema, respiratory (dyspnea, cough, and rhinitis) and/or gastrointestinal symptoms (abdominal pain, diarrhea, and meteorism) due to food consumption confirmed by positive SPTs with commercial food-allergens and/or fresh food and positive sIgE (>0.35 kU/L).

Thirty-nine patients with well-documented food symptoms and positive allergological workup were included in the study. BAT was positive in 29 patients. The mean age of the subjects with positive results of BAT was 15 (±10) years (range: 3–50 years). Seventeen patients (59% of the study sample) suffered from cow’s milk allergy. Almost all patients with cow’s milk allergy had positive skin prick tests to α-lactoalbumin, β-lactoglobulin, and casein (88%, 88%, and 94%, respectively). Along the same lines, elevated serum levels of specific IgE to α-lactoalbumin, β-lactoglobulin, and casein were observed in this group of patients (88%, 65%, and, 100%, respectively). As regards the group of patients with hen’s egg allergy, all subjects showed positive skin prick tests to both egg extracts (white and yolk) and elevated serum levels of specific IgE to egg white. Moreover, an IgE positivity to egg yolk was detected in 10 (83%) subjects of this group.

All patients underwent BAT before oral immunotherapy (OIT) initiation (time 0, T0) and started treatment with second-generation antihistamines (cetirizine, ebastine, or rupatadine) once a day because of concomitant allergic rhinitis. Therapy was continued for the entire OIT period.

After 3 months of starting protocol treatment (T1), BAT was performed for each patient.

The study was approved by the Institutional Review Board at the Fondazione Policlinico Universitario A. Gemelli IRCCS in Rome (ID3301) and performed in accordance with the Declaration of Helsinki.

The investigations were carried out following the rules of the Declaration of Helsinki of 1975, revised in 2013. All participants provided written informed consent, and minor’s parent or legal guardian gave consent to the study for under 18 patients. 

### 2.1. Skin Test

All patients underwent SPTs using commercial extracts and prick-by-prick (PBP) with fresh foods. The skin prick and PBP tests were performed according to standardized European protocols [13] with commercialized extracts of milk and egg proteins (Alk-abellò, Milan, Italy) and fresh foods (PBP method). Tests were considered positive when wheals were equal or larger than 3-mm compared with negative control. All SPTs were performed and read after 15 min according to EAACI guidelines [13].

SPTs with histamine (10 mg/mL) and saline solution were carried out as positive and negative controls, respectively.

### 2.2. Serological Tests

Measurement of specific IgE to cow’s milk proteins (α-lactoalbumin, β-lactoglobulin, and casein) and to hen’s egg proteins (white and yolk) was performed according to the manufacturer’s instructions (ImmunoCAP System, Phadia AB, Uppsala, Sweden).

Samples with specific IgE concentrations > 0.35 kU/L were considered as positive. Moreover, we assessed the value of serum total IgE (ImmunoCAP System, Phadia AB, Uppsala, Sweden).

### 2.3. Basophil Activation Test

The basophil activation test was performed using the Flow CAST^®^ assay (BÜHLMANN Laboratories, Schönenbuch, Switzerland) according to the manufacturer’s instructions. An aliquot of 0.05 mL of peripheral blood, from patients with allergy to egg or milk proteins, was incubated with allergens at the final concentration of 50 ng/mL (α-lactoalbumin, β-lactoglobulin, casein, egg white, and yolk) for 15 min at 37 °C along with monoclonal antibodies to human CD63 labelled with fluorescein isothiocyanate (anti-CD63 FITC) and human chemokine receptor CCR3 labelled with phycoerythrin (anti-CCR3-PE). Control conditions included a medium-only negative control, a positive control involving the cross-linking of the high-affinity Fc epsilon receptor (anti-FcεRI), and a positive control independent of FcεRI signaling, involving stimulation with N-Formylmethionyl-leucyl-phenylalanine (fMLP). CCR3 is constitutively expressed on eosinophils and basophils.

Flow cytometric acquisition with FACSCanto (BD Biosciences—Software FACSDiva, USA) was performed on flow cytometer working with a 488 nm argon laser diode (blue-green excitation light).

Results of BAT were presented as the percentage of CD63+ basophils. Results were considered positive when the difference within activated basophils of the patient with and without allergen (negative control) is greater than or equal to 15% [8].

The general sensitivity of the test, defined by the positivity for at least one specific allergen among those tested, was 74% for cow’s milk allergy and 73% for hen’s egg allergy before treatment with antihistamines. The sensitivity was reduced after the treatment (65% for cow’s milk allergy and 67% for hen’s egg, respectively). At baseline, the sensitivity of the test to α-lactoalbumin, β-lactoglobulin, and casein was 61%, 43%, and 70%, respectively, while the sensitivity of the test to egg white and yolk was 73% and 67%, respectively. Three months after antihistamine treatment, sensitivity to milk proteins significantly decreased to 48%, 35%, and 57%, respectively (*p* = 0.0032). A similar behavior was observed for the sensitivity values to hen’s egg proteins. Three months after antihistamine treatment, sensitivity to hen’s egg proteins decreased to 67% and 53%, respectively (*p* = 0.0572). 

### 2.4. Statistical Analysis

The sample was described calculating mean values and standard deviation for continuous variables, percentages for dichotomous or ordinal variables. Nonparametric tests used were the Wilcoxon test to compare averages and Mann–Whitney U test for independent samples.

Statistical analyses were performed using the IBM SPSS software package, version 20 (SPSS Inc., Chicago, IL, USA). A *p*-value < 0.05 was considered significant.

## 3. Results

In order to explore if basophil CD63+ expression was influenced by the use of antihistamines, the median percentages of BAT results for each specific culprit allergens (α-lactoalbumin, β-lactoglobulin and casein for cow’s milk, and egg white and yolk, respectively), before and after the administration of drugs, were compared with Wilcoxon signed-rank test, because of the heterogeneous distribution of the sample. No group presented significant change of average BAT percentages after treatment (Table 1).

**Figure 1:**
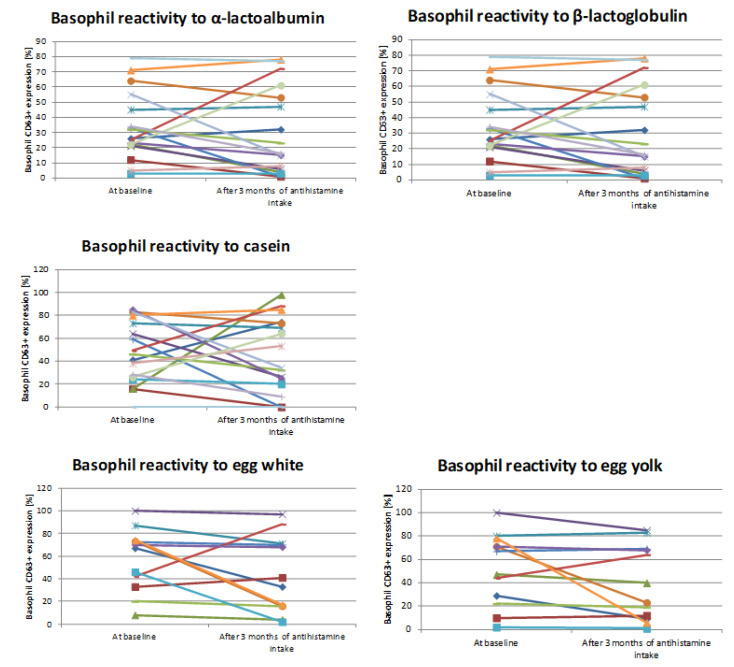
Basophil reactivity before and after therapy for each “specific allergen group.” Basophil CD63+ expression of each patient for single specific culprit allergen before and after 3 months of antihistamine treatment (Wilcoxon signed-rank test).

Individual basophil CD63+ expression for each specific culprit allergen is showed in Figure 1.

In study population, anti-FcεRI stimulation resulted in an intense increase in expression of basophil CD63. In addition, the stimulation with fMLP induced increase in the median percentage of CD63-positive cells. Three months after antihistamine treatment, the results of cell stimulation were not changed. 

**Figure 2:**
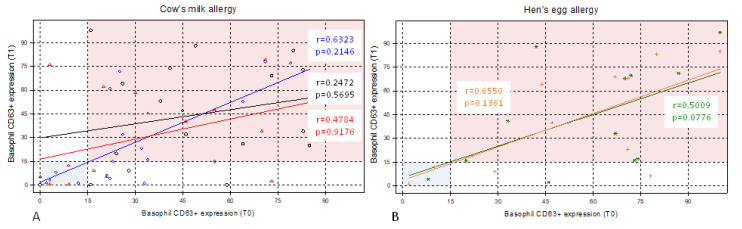
Pre- and post-treatment distributions of basophil CD63+ expression in cow’s milk allergy and hen’s egg allergy. Distributions of percentages of basophils with the expression of CD63 in (**A**) patients with cow’s milk allergy (n = 17) and (**B**) patients with hen’s egg allergy (n = 12) before (time zero, T0) and 3 months after antihistamine treatment (time one, T1). The left lower quadrant (blue area) represents the area in which a patient with cow’s milk or hen’s egg allergy tested negative in basophil activation test (BAT). (**A**): Blue diamonds indicate basophil CD63+ expression to α-lactoalbumin. Red triangles indicate basophil CD63+ expression to β-lactoglobulin. Finally, black circles indicate basophil CD63+ expression to casein. (**B**): Green stars indicate basophil CD63+ expression to egg white, orange plus indicate basophil CD63+ expression to egg yolk. r, Spearman’s correlation coefficient. *p*-value with Wilcoxon signed-rank test.

Plots were constructed for the distributions of percentages of basophils with the expression of CD63, which are shown in Figure 2, for patients with cow’s milk allergy and those with hen’s egg allergy, before and 3 months after antihistamine treatment. The left lower quadrant (blue area) represents the area in which a patient with milk or egg allergy tested negative in BAT.

The next step was grouping of patients according to single or multiple sensitization, maintaining the same stratification for single culprit allergen to explore if multiple sensitizations influences the change after antihistamine intake. Fourteen of 17 patients had at least one allergen sensitization to cow’s milk proteins, while 9 of 12 patients showed allergen sensitization to both egg’s proteins. Only the reactivity of basophils to casein, before the administration of antihistamines, was significantly different between the groups of patients (*p* = 0.0438, Mann–Whitney U test). This significant difference was not confirmed after administration of the drug.

## 4. Discussion

To the best of our knowledge, this was the first study, which focused on the influence of antihistamines on BAT results in cow’s milk and egg food allergy. The second-generation antihistamines used in this study (cetirizine, ebastine, and rupatadine) did not influence BAT results to specific culprit allergen. These drugs act as H_1_ blockers, competitive pharmacological antagonists at the H_1_ receptor, without effect on histamine release from storage sites, as basophils. 

According to Hoffman et al. [4], BAT can be used in all equipped laboratories to perform a flow-cytometric analysis to evaluate the CD63 expression on basophils after allergen-mediated activation of mast cells and basophils. In addition, a high specificity of BAT was demonstrated if compared with SPT and specific IgE as described in detail by Santos and Shreffler [3].

Moreover, BAT may be useful to identify patients with high risk of severe reaction to oral food challenge, because the basophil reactivity is often related to the severity of reaction [6,14,15,16].

In literature, a few studies focused on the impact of antihistamines on BAT, in particular in patients who are candidates to long period of treatment.

Sturm et al. performed two studies indicating that BAT is not influenced by antihistamines [9,17]. Wolanczyk-Medrala and coworkers evaluated the variation of BAT, 2 h after cetirizine intake, on patients with respiratory allergy to dermatophagoides pteronyssinus and pollens and showed that cetirizine did not influence the test sensitivity allowing the researchers to conclude that this diagnostic procedure can be performed in patients taking this antihistamine [12].

In our study, we performed BAT using single major allergens of cow’s milk (α-lactoalbumin, β-lactoglobulin, and casein) and food extracts of egg (white and yolk) to reduce the interference due to heterogeneous amount of component/s among extracts [18] and to increase the diagnostic accuracy [3,5,6,14].

Our study highlights the lack of interference of antihistamine intake on BAT results in cow’s milk and egg food allergy. In particular, the assumption of second-generation antihistamines (cetirizine, ebastine, and rupatadine) once a day for 3 months, due to concomitant allergic rhinitis, did not influence the reactivity of basophils even if patients were stratified according to specific culprit allergens (α-lactoalbumin, β-lactoglobulin and casein for cow’s milk, and egg white and yolk, respectively).

Likewise, basophil CD63+ expression was not modified by oral intake of antihistamines even if patients were stratified according to single or multiple sensitizations.

This study has some limitations. First, the retrospective and monocentric nature of the study, which describes a useful diagnostic tool for food allergy. Moreover, the number of enrolled patients was limited, partially due to high selective inclusion criteria. The design of the study was focused only on patients with food allergy, and concomitant allergic rhinitis requiring antihistamine therapy, undergoing OIT and, therefore, did not include a comparison with a control group. Furthermore, due to the observational nature of the study, we performed BAT according to the manufacturer’s recommendations without the evaluation of dose–response stimulation of basophils. Finally, we do not know if the extension of the time frame of primary outcome beyond the first 3 months had impact on basophil CD63+ expression in our studied population.

Nevertheless, according to our knowledge, this was the first study in literature assessing the effects of oral antihistamines on basophil reactivity in cow’s milk and egg food allergy. Our data strengthen the inclusion of BAT in the diagnostic work out of allergy since differently from in vivo tests, BAT is a functional test that can be performed when patients were taking antihistamine drugs.

Further prospective studies are necessary to explore changes in basophil response during treatment with antihistamines in food allergy and to amplify its clinical applications as in vitro assay.

In conclusion, BAT can be performed in patients taking antihistamines.

## Figures and Tables

**Table 1:** Basophil reactivity before and after therapy for each “specific allergen group.”

Total Sample	N of pts	29		
Group	N of pts (% of group)	17		
AR ^‡^ (perennial allergens)	N of pts (% of group)	10 (59)		
AR ^‡^ (seasonal allergens)	N of pts (% of group)	7 (41)		
cetirizine	N of pts (% of group)	12 (70)		
ebastine	N of pts (% of group)	3 (20)		
rupatadine	N of pts (% of group)	2 (10)		
Mono-sensitization	N of pts (% of group)	3		
Poly-sensitization	N of pts (% of group)	14		
Basophil CD63+ expression		Baseline	After 3 months	*p* value *
egg white	Median (IQ range ^?^)	26 (22–25)	16 (5–57)	0.2146
egg yolk	Median (IQ range ^?^)	20 (3–51)	12 (2–53)	0.9176
casein	Median (IQ range ^?^)	46 (25–77)	34 (15–74)	0.5695
Group	N of pts (% of group)	12		
AR ^‡^ (perennial allergens)	N of pts (% of group)	9 (75)		
AR ^‡^ (seasonal allergens)	N of pts (% of group)	3 (25)		
cetirizine	N of pts (% of group)	10 (80)		
ebastine	N of pts (% of group)	1 (10)		
rupatadine	N of pts (% of group)	1 (10)		
Mono-sensitization	N of pts (% of group)	3		
Poly-sensitization	N of pts (% of group)	9		
Basophil CD63+ expression		Baseline	After 3 months	*p* value *
egg white	Median (IQ range ^?^)	69 (35–74)	37 (16–71)	0.0776
egg yolk	Median (IQ range ^?^)	57 (24–76)	32 (10–69)	0.1361

BAT: basophil activation test. AR ^‡^: allergic rhinitis. IQ range ^?^: interquartile range. * :change of mean percentages of BAT after treatment (Wilcoxon signed-rank test).

## Data Availability

No new data were created or analyzed in this study. Data sharing is not applicable to this article.
